# Supplementary material for: The promotive role of lncRNA MIR205HG in proliferation, invasion, and migration of melanoma cells via the JMJD2C/ALKBH5 axis
Source: PLoS One. 2024 Jan 22;19(1):e0290986. doi: 10.1371/journal.pone.0290986 (PMC10802967; doi:10.1371/journal.pone.0290986)

Unprocessed western blot related to figure 3D

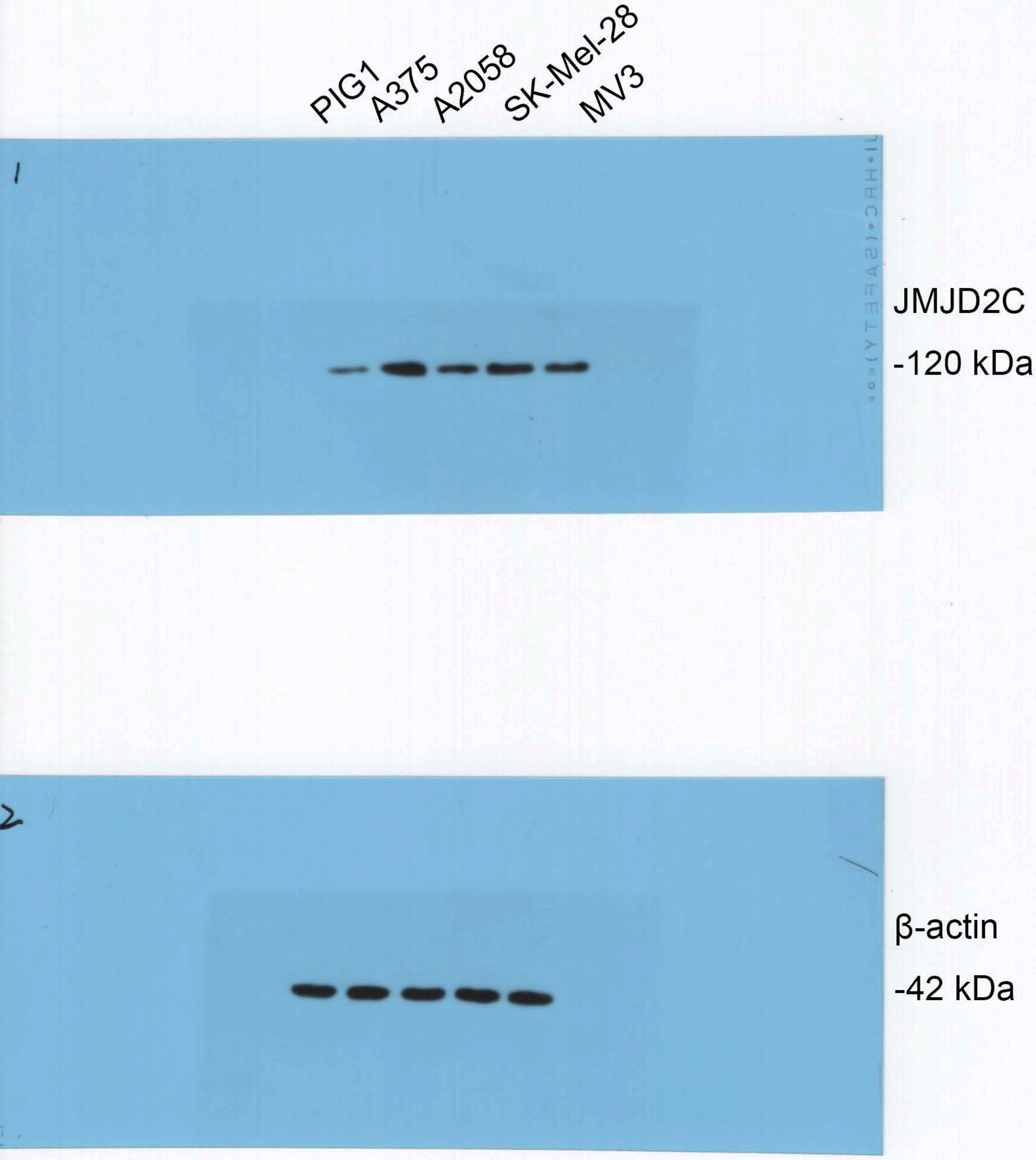

Unprocessed western blot related to figure 3D

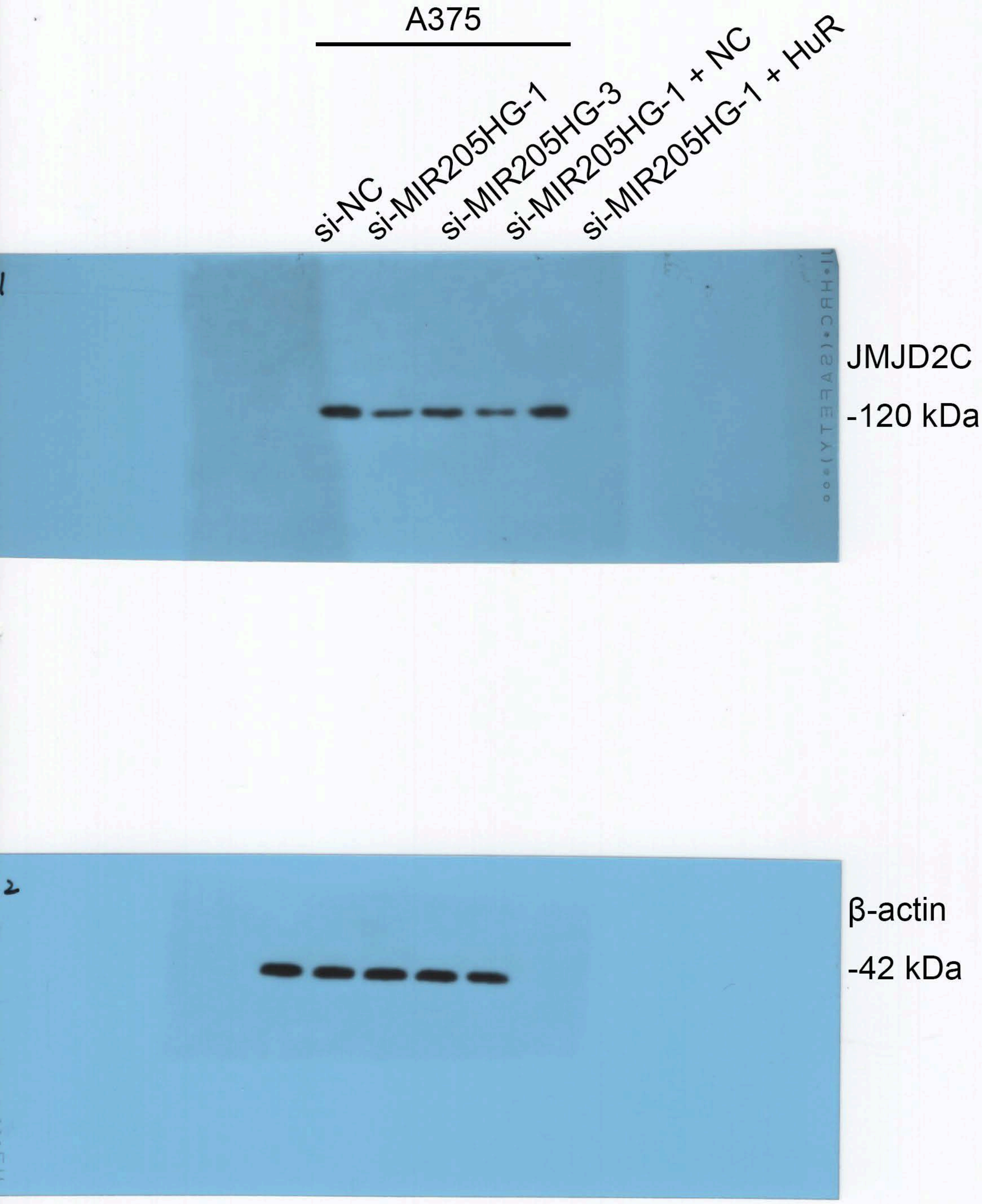

Unprocessed western blot related to figure 3D

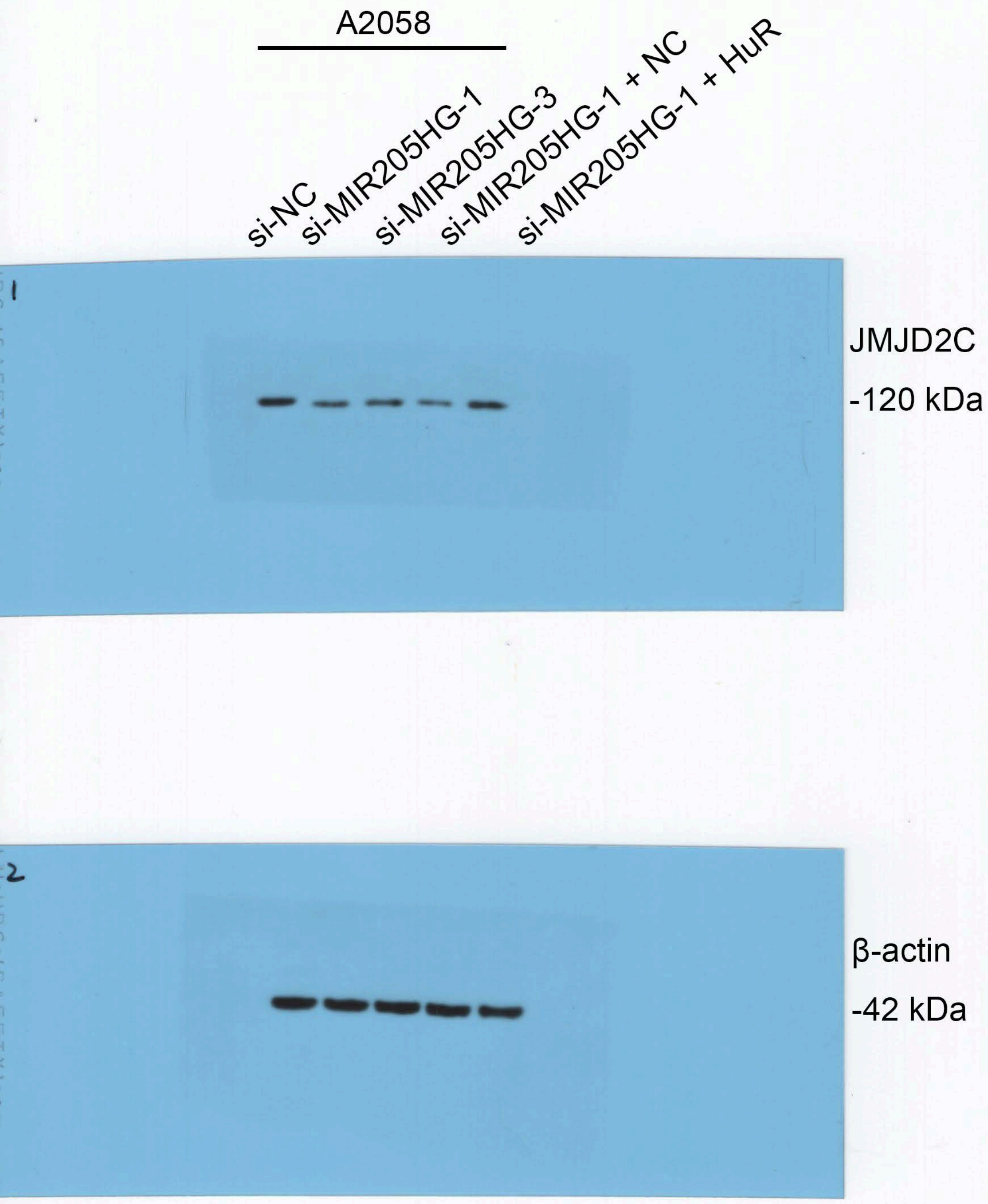

Unprocessed western blot related to figure 3G

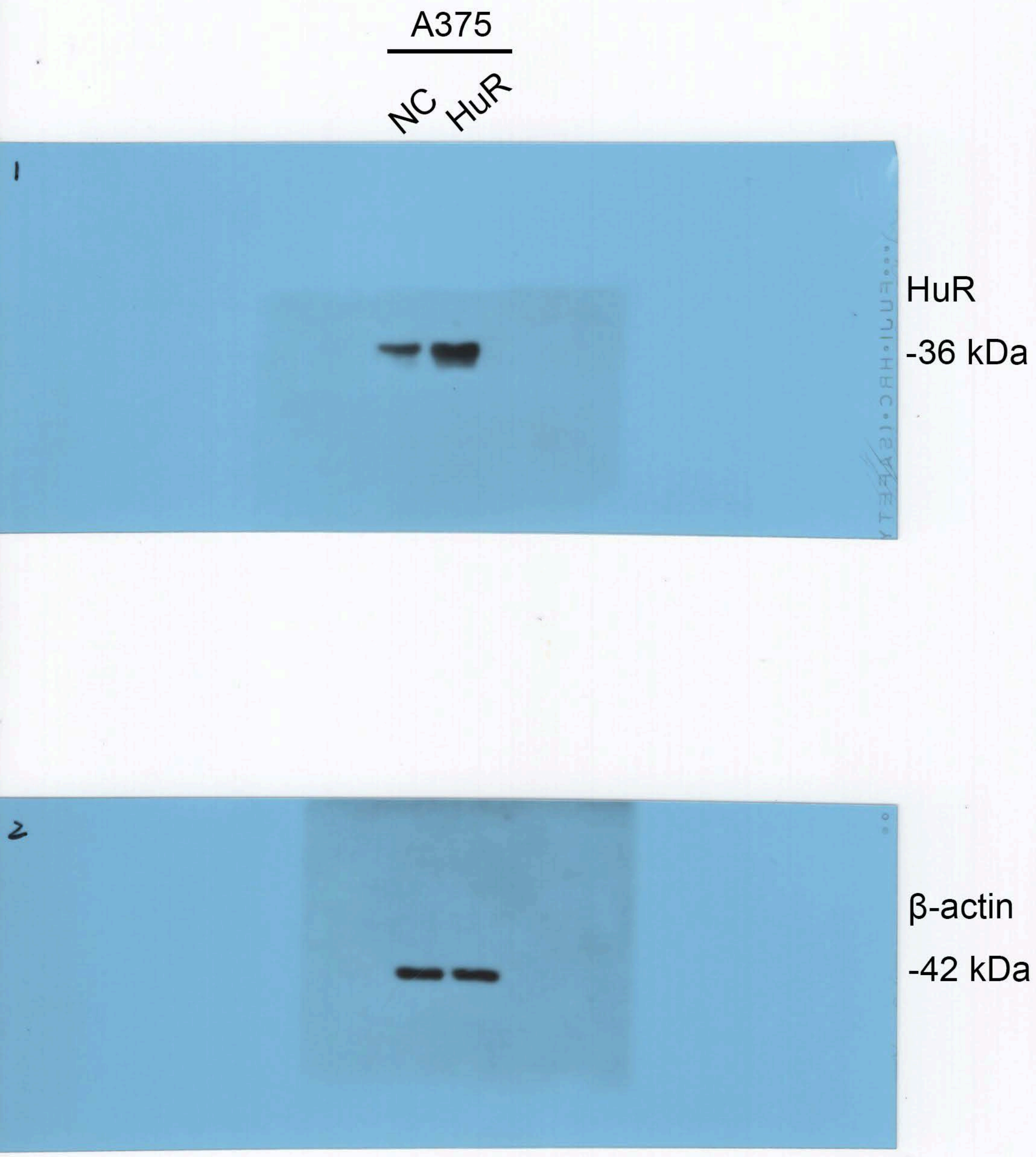

Unprocessed western blot related to figure 3G

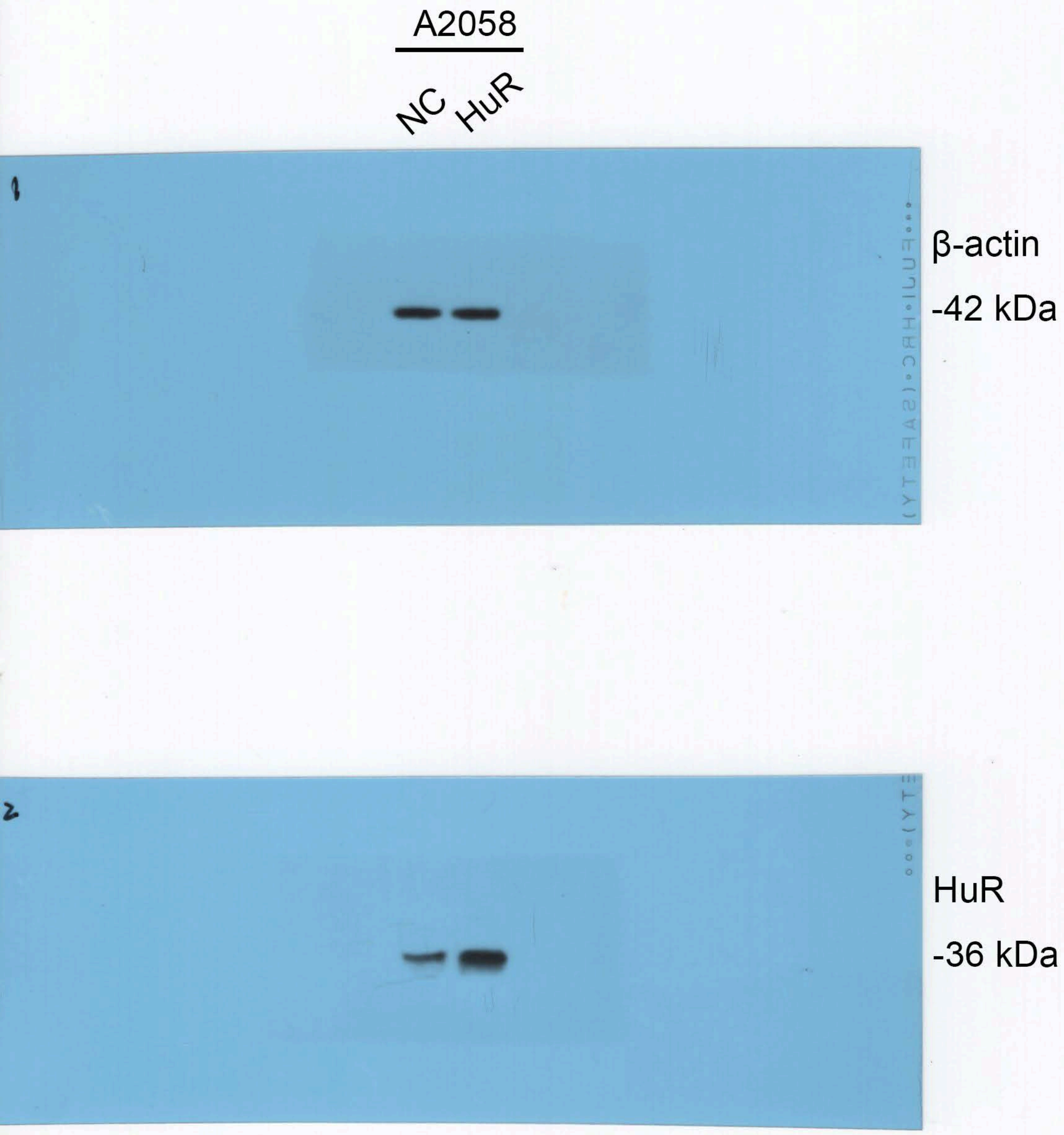

Unprocessed western blot related to figure 4B

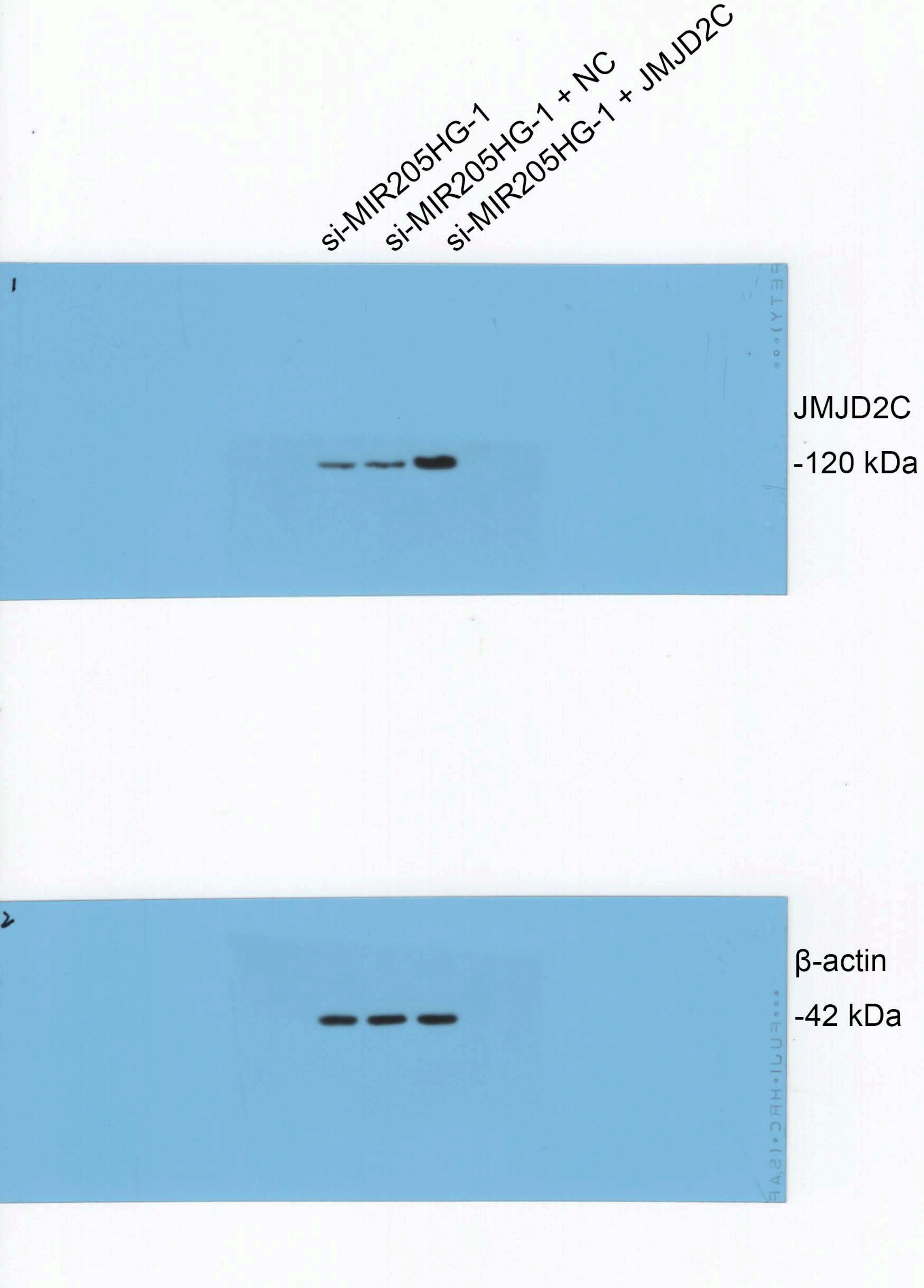

Unprocessed western blot related to figure 7G

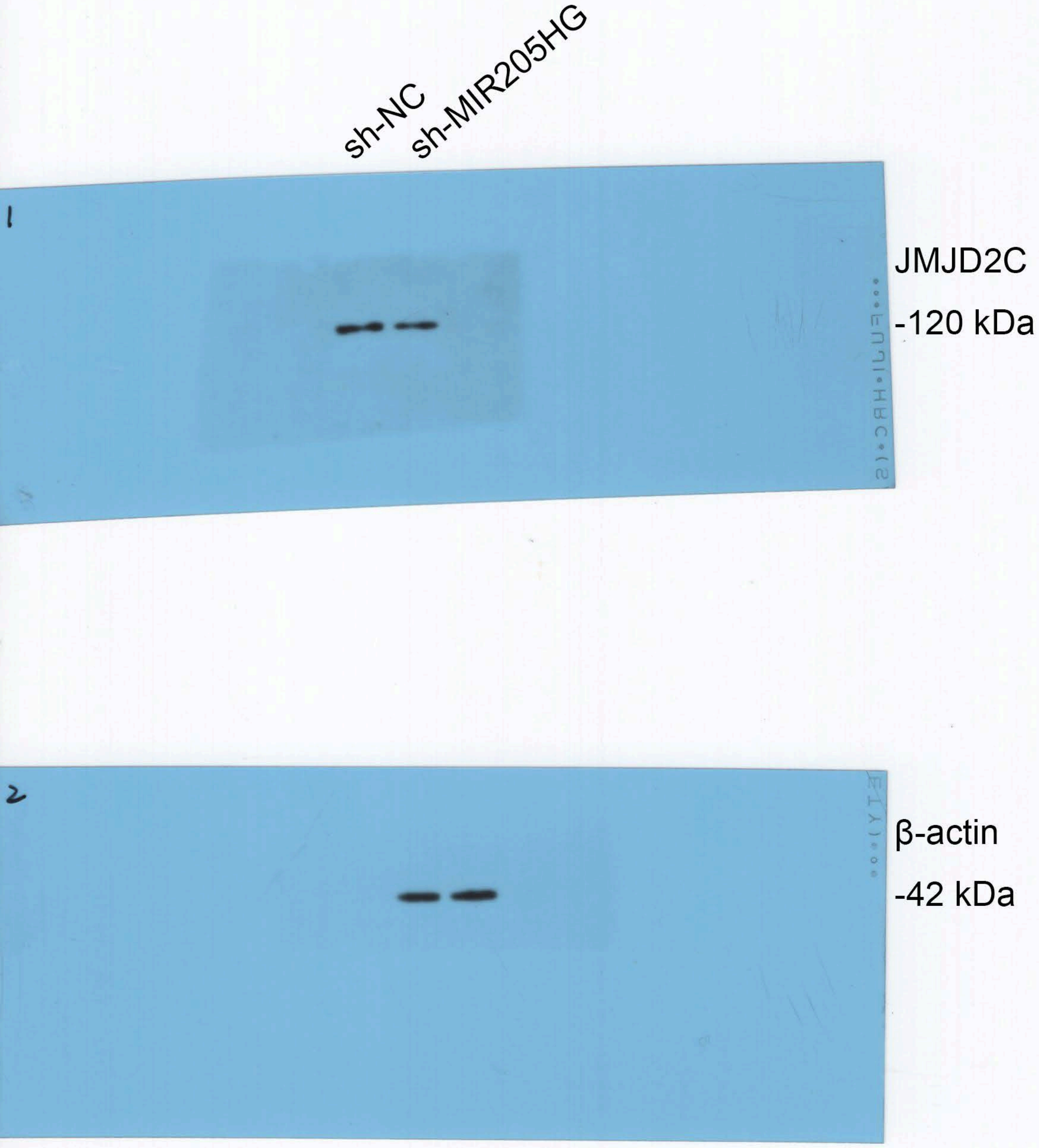

Supplement: S1 Raw images — (PDF) [file pone.0290986.s001.pdf]
